# Systemic Sarcoidosis Mimicking a Behavioural Variant of Frontotemporal Dementia

**DOI:** 10.1155/2015/409126

**Published:** 2015-09-09

**Authors:** Anne De Maindreville, Line Bedos, Serge Bakchine

**Affiliations:** Department of Neurology and CMRR, Hôpital Maison Blanche, University of Reims Champagne-Ardenne, 45 rue Cognacq Jay, 51100 Reims, France

## Abstract

Among rare neurological manifestations, a progressive dementia may exceptionally be the revealing clinical feature of a sarcoidosis. Diagnosis may then be difficult, especially when systemic signs are missing or latent, with a risk of therapeutic delay. We report the first case of sarcoidosis mimicking a frontotemporal dementia. A 53-year-old man presented with a dementia clinically suggestive of frontotemporal dementia, progressing slowly for about 2 years. However, MRI revealed unusual aspects, mainly large areas of T2/FLAIR hypersignal within temporal regions and cerebellum, with nodular leptomeningeal and juxtacortical Gadolinium enhancement. The patient was finally diagnosed with a systemic sarcoidosis. We discuss the differential diagnosis based on MRI aspects and review the literature on the clinical, biological, and imaging features of sarcoidosis presenting with dementia. This case demonstrates that brain imaging remains mandatory in the exploration process of a patient with dementia. Although the patient presented with rather typical features of a behavioural variant of frontotemporal dementia, the MRI aspect was the key exploration that leaded to the diagnosis.

## 1. Introduction

Clinicians may be confronted with patients presenting with a dementia due to a general disease clinically mimicking a neurodegenerative dementia. Diagnosis may be very difficult when systemic signs are missing. In this paper, we report on a patient suffering from sarcoidosis that presented clinically as a frontotemporal dementia. This case highlights the fact that MRI remains mandatory even in typically looking forms of dementia.

## 2. Case Presentation

A 53-year-old mechanic was admitted for the exploration of a progressive dementia. He had no significant past medical history. Two years earlier, he had started complaining of asthenia, sleeping difficulties, attention difficulties, and impairment of recent memory. His relatives described him becoming progressively more irritable and apathetic. Patient's general practitioner suspected first a depression, and patient was treated 14 months with SSRI antidepressants without clear benefit. As symptoms worsened progressively, the patient was then referred to a neurologist who diagnosed a behavioural variant of frontotemporal dementia (bvFTD). Due to the importance of cognitive and mostly behavioural symptoms, patient was referred to our centre. Clinically, the patient presented with apathy, reduction of speech, loss of empathy towards relatives, clear social disinhibition, gluttony, and perseverative and stereotyped behaviour. Physical examination showed no abnormality. The general status was preserved. The mini mental status examination (MMSE) score was 27/30. The neuropsychological evaluation showed a global cognitive efficiency in the mean (WAIS-III Global IQ: 99), with a significant dissociation between the verbal (104) and the performance (95) IQ scores. Verbal working memory was impaired. Grober-Buschke's FCSRT-16 test showed impaired immediate and delayed recall performances, almost totally compensated by semantic cueing. Testing of executive functions (Stroop Test; Wisconsin Card Sorting Test; verbal and graphic fluencies; Trail Making Test; Luria's graphic and gesture series; subtests from WAIS III: Image Picture Arrangement, Similarities, and Digit Symbol Coding Test) showed reduced mental flexibility, perseverative behaviour, and a global reduction of speed of information processing. There were no other significant impairments beside an important verbal disinhibition interfering with the testing.

**Figure 1:**
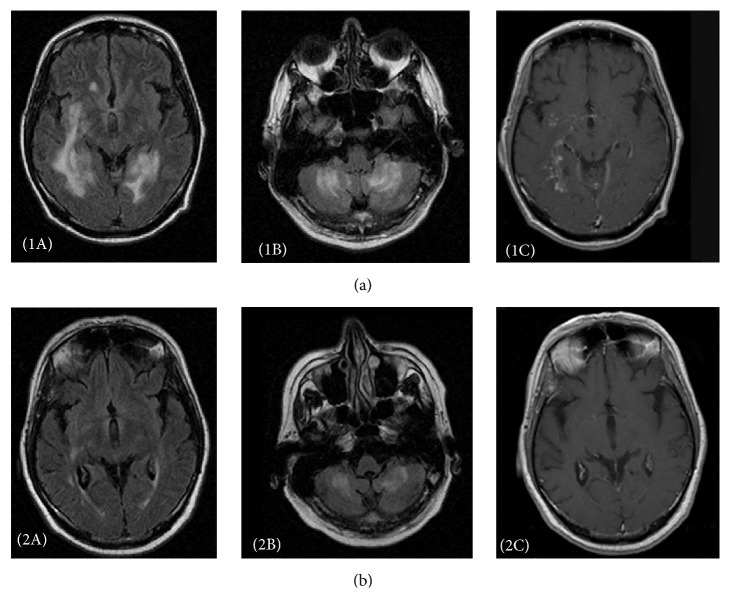
(a) Pretreatment aspects. T2 FLAIR sequences showing disseminated and confluent subcortical hyperintensities within frontoorbital, insular, and internal temporal regions (panel 1A) and cerebellum (panel 1B). Gadolinium injection (panel 1C) reveals nodular enhancement within the same regions and in leptomeningea. ((b) panels 2A, 2B, and 2C) Posttreatment aspects within approximately the same slices, showing an important regression of pathological aspects observed previously.

Despite clinical and neuropsychological features highly suggestive of a bvFTD, this diagnosis was considered as very unlikely as brain MRI did not show the typical features (frontal and temporal polar atrophy) of this syndrome but revealed instead aspects very unusual in FTD with confluent areas of T2/FLAIR subcortical hyperintensities located in right frontoorbital and insular regions but involving mostly both internal temporal white matter and cerebellar hemispheres (Figure 1(a) panels (1A) and (1B)). Gadolinium injection revealed nodular enhancement within the same regions and in leptomeningea (1C). A thoracoabdominal CT scan (without and with contrast injection) showed no significant abnormalities beside a mild nonspecific hepatomegaly. Blood work-up revealed lymphopenia (600/mm^3^) and slightly increased sedimentation rate with normal serum immunoglobulin electrophoresis. Serologies for HIV1 and HIV2, hepatitis B and hepatitis C, Lyme, syphilis, brucellosis, and rickettsia were negative. There was no serological indication of either an immunological disease or a paraneoplastic syndrome. Tuberculin skin test was negative.

CSF examination showed a normal cell count and a mild hyperproteinorachia (628 mg/L) with slightly elevated levels of immunoglobulin G and A, without oligoclonal immunoglobulin bands, and normal ACE level. Search for Koch's bacillus (PCR and culture),* Cryptococcus* (culture and antigen detection), and* Tropheryma whipplei* (PCR) was negative. Levels of CSF biomarkers (14-3-3, neuron specific enolase, tau and phosphorylated tau, and beta-amyloid) were within normal range. Bronchial endoscopy showed a nonspecific inflammatory liquid (testing for Koch's bacillus was negative). Minor salivary gland biopsy was negative. Duodenal biopsy analysis was negative for* Tropheryma whipplei*.

At this stage, both the patient and his family requested a rest in the explorations. Control brain MRI and gallium scintigraphy were scheduled for the following weeks; however, the patient was readmitted only 4 months later due to his behavioral difficulties. By this time, both cognition and behaviour had continued deteriorating (MMSE was 19/30 at readmission). Brain MRI showed increased high intensity lesions in temporal and cerebellar white matter, with patchy nodular enhancement after contrast administration. Gallium scintigraphy showed gastric and hepatic hyperfixations. Targeted hepatic and gastric biopsies revealed multiple nonnecrotizing granulomas composed of lymphocytes, epithelioid histiocytes, and giant cells, allowing a definite diagnosis of sarcoidosis. Patient was started on prednisone 1 mg/kg/day. A year later, cognitive and behavioural symptoms stopped worsening but remained stable, despite a significant reduction of high signal intensity lesions on control MRI (Figure 1(b) panels (2A) and (2B)). Enhancement after contrast administration was minimal, with complete disappearance of the nodular enhancing lesions (Figure 1(b) panel (2C)). Patient was then lost to follow-up. He died eighteen months later of a myocardial infarction. In the meantime, his behaviour, although still impaired, has not worsened anymore based on the judgement of his general practitioner. No autopsy was performed.

## 3. Discussion

The patient presented clinically with a progressive dementia with neuropsychological and behavioural features mimicking a frontotemporal dementia. FTD is a common group of clinical syndromes of young onset (<65 years) dementia. Four main clinical variants are recognized, a behavioural variant (bvFTD) and 3 variants with primary progressive aphasia (semantic, logopenic, and nonfluent/agrammatic variants). With respect to aetiology, FTD syndrome covers a rather heterogeneous set of neurodegenerative diseases, which share a circumscribed involvement of frontal and anterior temporal lobes [1]. In the bvFTD variant, which is the most frequent, personality and behavioural changes are the revealing features and remain predominant in the course, progressively associated with impairment in executive functions and in language quality. The disease progression is usually rather slow over years. FTD-like syndromes due to nondegenerative diseases have been very rarely reported. In this case, the brain MRI was crucial to exclude a typical FTD as it showed very atypical aspects leading us to discuss other diagnoses compatible with these images.

CNS tuberculosis was considered first because of the diffuse nodular meningeal enhancement. However, meningeal enhancement is usually more pronounced in the basal cisterns and associated with tuberculomas, which may appear as hyperintense T2-weighted images with homogeneous nodular enhancement (noncaseating tuberculomas) or isointense to markedly hypointense with a rim enhancement (caseating tuberculomas) [2]. Here, although a diffuse nodular meningeal enhancement was present, it was not predominant in the basal cisterns, and no aspect of tuberculoma was observed. The clinical picture was not suggestive, with slow evolution and absence of typical symptoms (meningismus, headache, fever, cranial nerves palsies, and altered general status), and search for Koch's bacillus was negative (both culture and PCR).

This case could also evoke a primary vasculitis of the CNS, mainly Wegener's granulomatosis. However, it rarely presents with cerebral and meningeal involvement. The clinical picture consists of headache, stroke-like episodes, and more rarely acute/subacute encephalopathy. MRI findings variously include cerebral granulomatous lesions (often resulting from contiguous invasion from nasal or orbital disease), meningeal thickening, vascular lesions, cerebral atrophy, and nonspecific inflammatory white matter lesions [3]. In this case, the patient presented no sign of respiratory tract disease and no orbital, cutaneous, cardiac, or renal (glomerulonephritis) involvement, which are common manifestations of Wegener's disease. The patient tested negative for ANCA, and the nodular pattern of the meningeal enhancement was atypical for Wegener's granulomatosis.

We searched also for Whipple's disease. This multisystemic infectious disease, caused by* Tropheryma whipplei*, is usually characterized as a gastrointestinal and rheumatological disorder, but neurological manifestations are present in 10 to 43% of patients [4]. An isolated neurological presentation is rare (about 5%). Patients with neurological involvement present quite often cognitive dysfunction, psychiatric features, progressive supranuclear ophthalmoplegia, and altered level of consciousness. Dementia was reported in only one patient out of a series of 17 with confirmed Whipple's disease [4]. Brain MRI may be normal or show nonspecific aspects as multiple nodular enhancing lesions (often in mesial temporal lobes) or solitary space occupying lesion. CSF analysis usually shows hyperproteinorachia and/or elevated white cells. PCR for* Tropheryma whipplei* in the CSF is considered as most performing diagnostic test. Although our case exhibited some features compatible with the diagnosis, PCR for* Tropheryma whipplei* in the CSF and on the duodenal biopsy was negative.

Our case was less suggestive of a primary CNS lymphoma. Usually, patients present with acute/subacute encephalopathy (with or without fever), signs of intracranial hypertension, or focal neurological deficits progressing rapidly. MRI shows usually a solitary supratentorial mass lesion but other aspects may be encountered as meningeal or periventricular enhancement [5]. We found one case revealed by a progressive dementia in an 81-year-old woman, with nonspecific cortical and subcortical atrophy on MRI, in whom the diagnosis was made after autopsy [6]. In our case, CSF immunophenotyping was not supporting the diagnosis, which was finally excluded by histological evidence.

Finally, our case is illustrative of the very rare forms of sarcoidosis revealed by a dementia syndrome. To our knowledge, it is the only reported case mimicking FTD. Neurologic symptoms are rather rare (about 10%) in sarcoidosis and sometimes are the only manifestation (10−17%) [7, 8]. Neurologic involvement is usually revealed (in decreasing order of frequency) by cranial-nerve palsies, headache, ataxia, cognitive dysfunction, weakness, or seizures. Dementia is very rare: among a series of 50 cases of neurosarcoidosis, only 5 cases of dementia syndrome and 2 cases of isolated amnesic syndrome were reported [9]. There are also a few single cases [10–13]. The pattern of cognitive impairment is variable, such as evolution rate. Mood and behavioural symptoms are often present but are rarely predominant [13]. In elderly patients, a progressive onset can mimic a degenerative dementia [11]. The diagnosis of sarcoidosis may then be very difficult when systemic signs are missing or latent.

Some biological abnormalities may be supportive of the diagnosis (lymphopenia, inflammatory signs, polyclonal hypergammaglobulinemia, hypercalcemia, elevated ACE, elevated beta-2 microglobulin, CSF hyperproteinorachia with lymphocytic pleocytosis, and increased ACE level), especially when associated with a negative tuberculin skin test in a previously vaccinated patient [14]. However, many of these biological abnormalities are nonspecific and may be absent. Brain MRI may provide very important clues. Typically it shows nodular or diffuse leptomeningeal enhancement, more pronounced in the basal cisterns [7, 8, 10]. However, MRI may be normal (20%) or show nonspecific or pseudotumorous lesions [8, 15]. Finally, diagnosis relies mostly on the histological demonstration of typical noncaseating epithelioid-cell granulomas. Imaging techniques (CT or MRI, gallium scintigraphy, and in some cases 18 FDG PET) play an important role by selecting the most easily accessed involved organs and guiding biopsies [14, 15]. In this case, although the clinical presentation was compatible with usual criteria for bvFTD [1], some biological signs appeared unusual (lymphopenia and elevated proteinorachia). Brain MRI was essential, as it showed aspects not compatible with a neurodegenerative disease. Finally, in the absence of obvious systemic lesions, gallium scintigraphy was decisive by showing sites with abnormal hyperfixation and allowing targeted biopsies, which showed the typical nonnecrotizing granulomas. We believe that the peculiar clinical picture observed in this case resulted from particularly important damage of the anterior temporal regions with frontal subcortical disconnection. The treatment of choice in neurosarcoidosis relies on corticosteroids, although some more aggressive options have been proposed, as evolution may be fatal [14]. In this case, contrary to others [10], a one-year treatment failed to improve cognition and behaviour, although MRI showed a significant improvement of lesions. We think very unlikely this evolution could be due to the simultaneous occurrence of a neurosarcoidosis and an authentic degenerative FTD. Apart from neuroimaging aspects, the main argument against this hypothesis is the absence of worsening since the treatment of sarcoidosis when patient's condition would have irremediably declined in the case of true FTD. We consider instead that this relative failure may be due to a long-lasting disease and delayed treatment. A further improvement could not be assessed due to loss of follow-up.

We think that further reports of such cases are important, as underlying diseases may be potentially curable, which is not presently possible for neurodegenerative frontotemporal dementias.
